# New Drugs, Appliances, and Things Medical

**Published:** 1891-05-16

**Authors:** 


### NEW DRUGS, APPLIANCES, AND THINGS MEDICAL.

[All preparations, appliances, novelties, etc., of which a notice is desired, should be sent for The Editor, to care of The Manager, 140, Strand, London, W.C.]

### MALT EXTRACT, "STANDARD" BRAND.

This is a remarkably good specimen of malt extract. The analyses forwarded to us show the extremely high percentage of the diastatic ferment. We have made control experiments, and quite agree that this extract is one of the most active we have examined. Besides the high percentage of diastase, there is a large amount of "Maltose" present. The ash after incineration yielded good quantities of alkaline and earthy phosphates, all of which tend to aid nutrition. The extract is also made up with codliver-oil. The result is a homogeneous thick fluid which very effectually disguises the taste and smell of the oil, and so will enable those who are unable to take the oil *au naturel* to find no difficulty in digesting their daily quantum of fat. The careful and scientific feeding of invalids is now well understood to be at least half the treatment of disease, and those requiring reliable preparations may make use of the above with every confidence. Offices, 23 to 25, Billiter Street, E.C.

